# Primary hyperthyroidism complicated by primary hyperparathyroidism: a case report and literature review

**DOI:** 10.3389/fonc.2025.1524367

**Published:** 2025-03-28

**Authors:** Hongpeng Guo, Jie Lv, He Zhang, You Li, Xinghe Pan, Junjie Zhang, Chenglin Sun

**Affiliations:** ^1^ Department of General Surgery, The Second Hospital Affiliated to Shenyang Medical College, Shenyang, China; ^2^ Department of Clinical Laboratory Center, Xinjiang Medical University Affiliated Tumor Hospital, Urumqi, China; ^3^ Department of Orthopedics, Tongliao People’s Hospital, Tongliao, Inner Mongolia, China; ^4^ Department of Pathology, Central Hospital Affiliated to Shenyang Medical College, Shenyang, Liaoning, China

**Keywords:** pH, PHPT, Graves’ disease, MDT, thyroid storm

## Abstract

**Background:**

The clinical occurrence of primary hyperthyroidism (PH) combined with primary hyperparathyroidism (PHPT) is exceedingly rare. There remains considerable debate regarding the necessity of iodine use prior to surgery for hyperthyroidism and whether thyroid function should be normalized before proceeding with the operation. Furthermore, the decision on whether to perform total parathyroidectomy or subtotal parathyroidectomy due to parathyroid hyperplasia must be based on a comprehensive assessment by a multidisciplinary team (MDT).

**Case presentation:**

Herein, we report a rare case of concurrent PH, caused by Graves’ disease(GD), and PHPT. Through the collaboration of a MDT, we developed a personalized preoperative preparation and surgical plan for the patient, successfully managing the postoperative complications.

**Conclusion:**

Clinicians should maintain a high level of suspicion for PHPT in hyperthyroid patients with hypercalcemia. Additionally, the decision regarding the preoperative use of iodine, the normalization of thyroid function before surgery, and the surgical approach to parathyroid hyperplasia should be determined through effective preoperative assessment, imaging studies, and MDT collaboration. This strategy allows for the formulation of individualized treatment plans, mitigating the risks of postoperative hyperthyroid crises, recurrence of PHPT, and permanent parathyroid insufficiency.

## Introduction

Hyperthyroidism is a hypermetabolic disorder characterized by abnormally elevated levels of thyroid hormones in the body, typically classified into three categories: primary, secondary, and functional adenoma (1). Among these, primary hyperthyroidism is the most prevalent, often resulting from Graves’ disease(GD), and is commonly observed in individuals aged 20 to 40 years. Patients typically present with diffuse bilateral thyroid enlargement accompanied by exophthalmos, hence the term “exophthalmic goiter.” (2). The etiology of primary hyperthyroidism (PH) remains unclear, although it is widely considered an autoimmune disorder. Clinical manifestations include thyroid enlargement, irritability, insomnia, hand tremors, heat intolerance, excessive sweating, moist skin, increased appetite coupled with weight loss, and other symptoms (3).

Primary hyperparathyroidism (PHPT) is a metabolic disorder characterized by excessive secretion of parathyroid hormone, primarily manifesting as an imbalance in calcium and phosphorus metabolism (4). The most common cause of PHPT is parathyroid adenoma, accounting for 85% of cases, followed by parathyroid hyperplasia, which constitutes approximately 12%, while the incidence of parathyroid carcinoma is exceedingly rare (5, 6). This condition predominantly affects the middle-aged and elderly population. It presents as autonomous hyperfunction of the parathyroid glands, leading to elevated blood calcium levels and decreased phosphorus levels. Clinical symptoms are diverse, including skeletal issues such as bone pain and fractures, as well as urinary symptoms like kidney stones (7).

PH and PHPT are both common endocrine disorders; however, their concurrent occurrence is exceedingly rare. Here, we report a case involving a female patient with PH complicated by PHPT. The patient presented with severe hyperthyroid symptoms preoperatively, and her thyroid mass was significantly enlarged, causing notable compression of the trachea. Postoperatively, the patient experienced a severe complication known as a hyperthyroid crisis, which further complicated her treatment. Through the collaboration of a multidisciplinary team (MDT), we developed a personalized preoperative preparation and surgical plan for the patient and successfully managed the postoperative complications. We present this case report along with a review of the relevant literature.

## Case presentation

### Patient information

A 35-year-old female patient was admitted in April 2024 due to “hyperthyroidism for 4 years, accompanied by neck swelling and exophthalmos for 2 years.” In September 2020, she presented with symptoms including increased appetite, weight loss, palpitations, heat intolerance, excessive sweating, polyuria, and thirst, without any obvious triggers. She was diagnosed with hyperthyroidism at a local hospital and treated with methimazole 10 mg once daily and bisoprolol 10 mg twice daily, which provided partial symptom relief. In February 2022, she discontinued the medication on her own, leading to a worsening of symptoms, including neck swelling, photophobia, tearing, a foreign body sensation, and heel pain. In June of the same year, she sought medical advice again, and the local hospital recommended surgical treatment, which she refused, opting instead to continue taking methimazole and bisoprolol. After stopping the medication again in June 2023, her symptoms persisted until she sought care at our hospital. To better understand the course of the patient’s condition, the key medical events are summarized in the following timeline (**Table 1**).

**Table 1:** Timeline of Key Medical Events.

Time	Event Description
September 2020	The patient first presented with symptoms such as increased appetite, weight loss, and palpitations, and was diagnosed with hyperthyroidism. She was treated with methimazole and bisoprolol.
February 2022	She discontinued the medication on her own, leading to a worsening of symptoms, including neck swelling, photophobia, and heel pain.
June 2022	She sought medical advice again, refused the suggestion of surgery, and continued medication treatment.
June 2023	She discontinued the medication again, and her symptoms persisted, ultimately leading to a referral to our hospital.
April 2024	She was admitted for MDT assessment and preoperative preparation (including iodine therapy, calcium-lowering treatment, etc.), and underwent total thyroidectomy and left inferior parathyroidectomy.
October 2024	Follow-up showed that thyroid function, PTH, and calcium-phosphorus levels were all normal.

MDT, multidisciplinary team; PTH, parathyroid hormone.

### Laboratory tests and imaging findings

**Figure 1:**
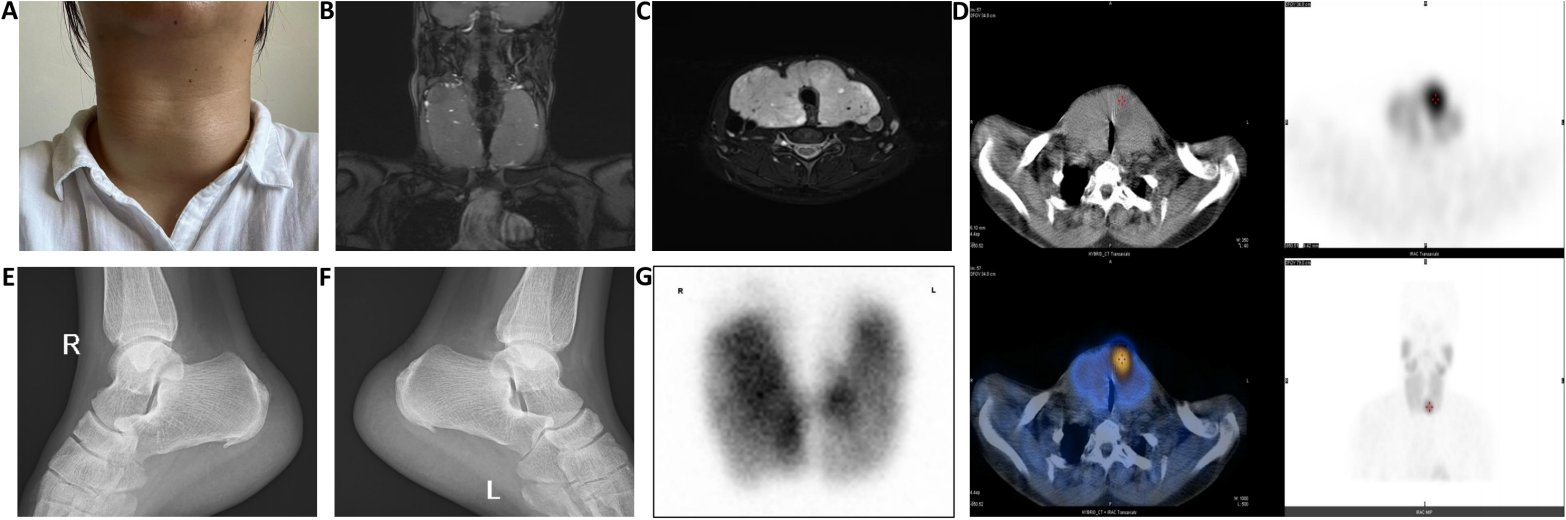
**(A)** The thyroid volume significantly decreased and became firm after two weeks of preoperative iodine treatment; **(B, C)** Thyroid MRI shows significant enlargement of the bilateral thyroid lobes and isthmus, with compression and narrowing of the trachea; **(D)** After intravenous injection of 370 MBq of ^99m^Tc-MIBI, imaging at 15 minutes shows normal positioning of both thyroid lobes with an enlarged shape, an area of abnormally increased tracer uptake is observed near the isthmus at the lower pole of the left lobe, while the remaining lobes show uneven tracer distribution, at 120 minutes post-injection, the area of increased tracer uptake near the isthmus at the lower pole of the left lobe persists, with significantly reduced tracer distribution in the rest of both lobes, no other areas of abnormal tracer concentration are observed within the field of view; **(E, F)** X-ray findings show bone hyperplasia of the right and left calcaneus; **(G)** After intravenous injection of 185 MBq of ^99m^TcO_4_^-^, planar thyroid imaging at 20 minutes shows the thyroid in a normal position with an enlarged shape, the distribution of the tracer within both lobes is unevenly increased, displaying multiple areas of increased and sparse tracer uptake, no abnormal tracer distribution is observed in the remaining scan field.

Upon admission, physical examination revealed: temperature of 36.8°C, pulse of 120 beats/min, respiratory rate of 21 breaths/min, blood pressure of 145/92 mmHg, with grade III thyroid enlargement, pronounced exophthalmos, and fine tremors in both hands when extended. Thyroid ultrasound showed diffuse lesions and multiple nodules (TI-RADS grade 3). Laboratory test results were as follows: thyroid-stimulating hormone (TSH) at 0.001 mU/L (reference range: 0.27–4.20 mU/L), free triiodothyronine (FT3) at 15.27 pmol/L (reference range: 2.8–7.1 pmol/L), free thyroxine (FT4) at 39.27 pmol/L (reference range: 11.5–22.7 pmol/L), and TSH receptor antibodies (TRAB) at 17.25 U/L (reference range: <1.75 U/L). Thyroid Magnetic resonance imaging (MRI) revealed significant enlargement of both lobes and the isthmus, increased T2 signal with heterogeneous intensity, and multiple mixed or slightly prolonged T2 signal nodules, the largest located at the upper pole of the right thyroid lobe measuring 2.3 cm, exhibiting long T1 and mixed T2 signals. The thyroid capsule remained intact bilaterally, while the trachea was compressed and narrowed (**Figures 1B, C**). Static thyroid scintigraphy indicated bilateral thyroid enlargement with unevenly enhanced uptake and multiple mixed nodules within both lobes (**Figure 1G**). X-ray of the calcaneus demonstrated bone hyperplasia in both the left and right calcaneus (**Figures 1E, F**). Additionally, further laboratory tests revealed a significantly elevated serum calcium level of 3.75 mmol/L (reference range: 2.2–2.9) and parathyroid hormone (PTH) at 321.2 ng/L (reference range: 15.0–65.0 ng/L). This crucial finding suggests possible parathyroid dysfunction, prompting us to conduct further parathyroid imaging (MIBI dual-phase method), which indicated a zone of increased radiotracer uptake near the lower pole of the left thyroid lobe, strongly suggesting the likelihood of a hyperfunctioning parathyroid lesion (**Figure 1D**).

### Therapeutic interventions

**Figure 2:**
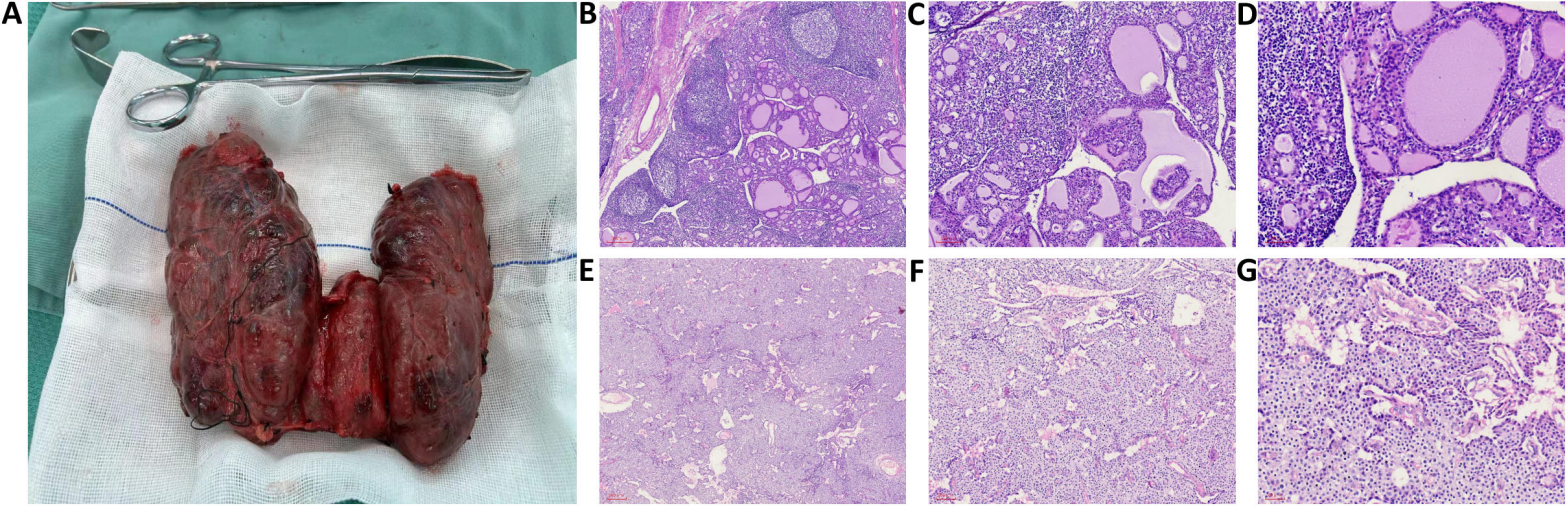
**(A)** Intraoperative findings showed diffuse, symmetric enlargement of the thyroid gland, approximately four times the size of a normal gland, the gland surface was smooth, with a red-brown, muscle-like cut surface, and a solid, moderate texture; **(B-D)** H&E-stained sections at magnifications of 50×, 100×, and 200×, respectively, show a microscopic view of proliferating tall columnar follicular epithelial cells with papillary projections extending into the follicular lumen. The colloid appears thinned with vacuolations, and the stroma shows congestion with lymphocyte infiltration and formation of localized lymphoid follicles; **(E-G)** H&E-stained sections at magnifications of 50×, 100×, and 200×, respectively, show a microscopic view of proliferation of chief cells, which appear polygonal with centrally located nuclei and coarse chromatin, with clear nuclear membranes. The cytoplasm is eosinophilic with some transparent vacuoles, and eosinophilic cells with granular cytoplasm are also observed.

The patient had experienced severe hyperthyroid symptoms for many years, with thyroid enlargement compressing the trachea, leading to significant psychological stress. Following discussions within a MDT, a comprehensive preoperative preparation and surgical plan was established. The patient was administered methimazole at 20 mg once daily and propranolol at 10 mg three times daily to manage hyperthyroid symptoms. Intravenous infusions of pamidronate disodium were given to reduce serum calcium levels. Due to the prolonged course of hyperthyroidism, oral prednisone was also prescribed at 10 mg twice daily to mitigate surgical stress and decrease the risk of thyroid storm postoperatively. Preoperative monitoring of the patient’s basal metabolic rate (BMR) was conducted, and medication dosages were adjusted accordingly to maintain thyroid function within a reasonable range while significantly lowering the BMR, with the heart rate stabilized at approximately 80–90 beats/min. Two weeks prior to surgery, the patient began taking Lugol’s solution (containing 8 mg of iodine per drop), orally three times a day, starting with 3 drops (0.05 mL/drop) and increasing by one drop each day until reaching a total of 16 drops, which was then maintained. Preoperative thyroid function tests revealed TSH at 7.671 mU/L (reference range: 0.27–4.20 mU/L), FT3 at 3.24 pmol/L (reference range: 2.8–7.1 pmol/L), FT4 at 6.89 pmol/L (reference range: 11.5–22.7 pmol/L), and serum calcium at 2.89 mmol/L (reference range: 2.2–2.9). Preoperative examination revealed that the thyroid volume had significantly decreased and become firm (**Figure 1A**). Under general anesthesia, a total thyroidectomy and left inferior parathyroidectomy were performed. Intraoperatively, the thyroid gland was observed to be diffusely symmetrically enlarged, approximately four times the size of a normal gland, with a smooth surface and a reddish-brown, muscular texture, classified as solid and of moderate consistency (**Figure 2A**). Intraoperative blood samples taken before and 10 minutes after excision revealed PTH levels of 279.3 ng/L and 38.4 ng/L, respectively, confirming complete removal of the diseased tissue. The surgery proceeded smoothly, but within 12 hours postoperatively, the patient experienced high fever, heart rate fluctuations between 140-180 beats/min, agitation, and vomiting. Management included administration of phenobarbital for sedation, physical cooling measures, oxygen supplementation, and intravenous glucose for caloric replenishment, alongside continued methimazole and propranolol for thyroid and heart rate control. Additionally, calcium and vitamin D were provided to address facial and perioral numbness. By postoperative day three, vital signs stabilized.

### Histopathological findings

Postoperative pathology revealed under microscopy that the thyroid follicular epithelial cells were tall columnar, with nuclei positioned at the base and no clear mitotic figures observed. The tall columnar epithelial cells exhibited hyperplasia, forming papillary structures that protruded into the follicular lumen. The colloid within the follicles appeared thin and lighter in color, with many vacuoles surrounding the colloid in some follicles, and a noticeable reduction in colloid content in others. The interstitium showed congested capillaries with significant lymphocytic infiltration, and localized lymph follicle formation was observed (**Figures 2B-D**). The pathological diagnosis indicated diffuse hyperplasia of the thyroid. Postoperative pathology of the parathyroid gland under the microscope shows parenchymal cell proliferation, predominantly consisting of chief cells. These cells are polygonal or round, with centrally located nuclei that exhibit coarse chromatin and well-defined nuclear membranes. The cytoplasm appears eosinophilic, amphophilic, or with a transparent vacuolated appearance. Changes in eosinophilic cells are also observed, with the cytoplasm showing a markedly eosinophilic granular texture and nuclei larger than those of other chief cells (**Figures 2E-G**). Pathological diagnosis: parathyroid hyperplasia.

### Follow-up and outcomes

Postoperatively, the patient was prescribed oral levothyroxine (LT4) for replacement therapy, with medication dosage adjusted during outpatient follow-up. Currently, six months after surgery, the patient’s thyroid function, PTH, blood calcium, and phosphorus levels are all within normal ranges.

## Discussion

The concurrent occurrence of HP and PHPT is clinically rare, despite both being common endocrine disorders. Studies indicate that some patients with PHPT also present with thyroid disorders, including goiter and thyroid dysfunction (8, 9). Wagner et al. found that the incidence of PHPT in patients with thyroid disease was 0.29%, compared to only 0.09% in those without thyroid disease (10). Similarly, Castellano et al. reported that among 238 PHPT patients, 21 had concurrent hyperthyroidism (11). However, the true coexistence rate may be underestimated. Research by Abboud et al. demonstrated that 13.5% of surgical PHPT patients also had hyperthyroidism, a figure higher than previously reported (12). We conducted a PubMed search for cases of PH combined with PHPT (**Table 2**) (9, 13–19). GD was the most common etiology of PH, while parathyroid adenoma was the most common cause of PHPT. Notably, two patients were found to have papillary thyroid carcinoma (PTC) during surgery. Some authors suggest that hypercalcemia may contribute to the carcinogenic effect on the thyroid; however, the relationship between hypercalcemia and PTC remains unclear (20). Further studies with larger sample sizes are needed. Additionally, patients with hyperthyroidism and PHPT should be vigilant for the possibility of multiple endocrine neoplasia (MEN), which typically arises from mutations in the same gene; thus, the simultaneous presence of hyperthyroidism and PHPT may be part of MEN syndrome (21). Prolonged hypercalcemia in hyperthyroid patients may influence parathyroid hormone secretion; in this case, it remains unclear whether the long-standing history of hyperthyroidism is associated with changes in parathyroid hormone levels. Therefore, it is still uncertain which condition developed first and whether there is a potential link between the two, warranting further investigation.

**Table 2:** Summary of reported cases with concurrent primary hyperthyroidism(PH) and primary hyperparathyroidism(PHPT).

Study (Year)	Age/Sex	Etiology	Clinical Presentation	Diagnostic	Surgical Approach	Postoperative Outcomes
Zhang et al. (9) (2022)	58/M	Toxic nodular goiter, Parathyroid adenoma	Weight loss, Neck enlargement, Hoarseness, Fatigue, tachycardia, Osteoporosis, Joint pain	US, CEUS, MRI, SPECT/CT, Blood tests, Histology	Subtotal thyroidectomy, MWA	Biochemical indicators rapidly recovered, and follow-up results remained stable
Musiałkiewicz et al. (13) (2024)	76/F	Toxic nodular goiter, Parathyroid adenoma	Massive goiter, Neck enlargement, Tracheal and esophageal compression	SPECT/CT, PTH, Radioisotope scanning, Blood tests, Histology	Not described	Not described
He et al. (14) (2015)	46/M	GD, Parathyroid adenoma, Papillary carcinoma	Polyphagia, Irritability, Progressive weakness, Epigastric pain, Nausea, Acute weight loss, Hypertension, Dry mouth, Mild thyroid enlargement	US, Radioisotope scanning, Blood tests, Histology	Total thyroidectomy and parathyroid adenoma resection	One year post-surgery, the patient is asymptomatic, with normal serum thyroxine and calcium levels
Yokomoto et al. (15) (2015)	52/F	GD, Hypercellular parathyroid tissues, Papillary carcinoma	Thirst, Nausea, Loss of appetite, Fatigue, Weight loss, Dry skin, Diffuse thyroid enlargement	US, CT,Radioisotope scanning, DXA,Blood tests, Histology	Thyroparathyroi-dectomy	One year post-surgery, calcium and PTH levels normalized, no recurrence of symptoms
Nagaki et al. (16) (2021)	12/F	GD, Parathyroid adenoma	Short stature, Cubitus valgus, Short neck, Irregular menstruation, Goiter	US, Radioisotope scanning, DXA,Blood tests, Histology	Subtotal parathyroidectomy	Postoperative Ca and iPTH levels normalized
Xiao et al. (17) (2002)	53/F	GD, Parathyroid adenoma	Heat intolerance, Excessive sweating, Palpitations, Hand tremors, Anterior neck swelling, Weight loss	US, Radioisotope scanning, Blood tests, Histology	Subtotal thyroidectomy, Parathyroid adenoma resection	Two weeks post-surgery, serum calcium, phosphate, I-PTH, and thyroid function were normal, with regular follow-up
Miani et al (18). (2003)	59/F	GD, Parathyroid adenoma, Parathyroid hyperplasia	Not described	US, Radioisotope scanning, CT, Blood tests, Histology	Subtotal thyroidectomy, Bilateral inferior parathyroidectomy	One year post-surgery, follow-up showed normal serum calcium, phosphate, and PTH levels
Tachibana et al. (19) (2012)	54/F	GD, Parathyroid hyperplasia	General fatigue, Acromegalic features, Diffuse goiter, Mild proptosis,Osteoporosis	US, CT, Radioisotope scanning, Blood tests, Histology	Thyroidectomy, Total parathyroidectomy	Biochemical indicators recovered, and bone markers improved at 17-month follow-up.

GD, Graves’ disease; M, male; F, female; primary hyperparathyroidism; US, ultrasound; CEUS, contrast- enhanced ultrasound; MRI, magnetic resonance imaging; SPECT/CT, single-photon emission computed tomography combined with computed tomogRaphy; MWA, microwave ablation; MNG, multinodular goiter; toxic nodular goiter; DXA, dual energy X-ray absorptiometry; TS, Turner syndrome.

Due to the masking effects of hyperthyroidism symptoms, PHPT is often diagnosed late in its course. The coexistence of both conditions complicates diagnosis, particularly in preoperative imaging, where thyroid pathology may hinder the accurate localization of parathyroid lesions (9).In this case, the patient was initially diagnosed with GD, characterized by years of hyperthyroidism, bilateral diffuse goiter, and exophthalmos. Thyroid ultrasound, MRI, and static thyroid imaging did not reveal any abnormalities in the parathyroid glands. The patient’s hypercalcemia posed a significant diagnostic challenge. While hyperthyroidism can indeed accelerate bone turnover, potentially leading to elevated blood calcium levels (22), PTH levels in hyperthyroid-induced hypercalcemia are typically suppressed, remaining normal or low. However, the markedly elevated PTH levels in this patient suggested a concurrent PHPT, which was subsequently confirmed through further imaging studies. Therefore, in clinical practice, special attention must be given to the possibility of PHPT in patients presenting with hyperthyroidism and hypercalcemia, ensuring timely and accurate diagnosis and treatment decisions.

The patient in this case meets the criteria for surgical treatment of GD. Firstly, the patient’s hyperthyroidism has persisted for four years, accompanied by significant goiter compressing the trachea, as well as moderate exophthalmos. Despite previous treatment with antithyroid medications, the poor efficacy and recurrent, severe symptoms prevented long-term control of the condition through medical management. Preoperative preparation for hyperthyroid patients is crucial. Currently, there is considerable debate regarding the routine use of iodine solutions (Lugol’s solution) for preoperative preparation in patients with GD. The 2016 ATA guidelines for the diagnosis and management of hyperthyroidism recommend preoperative iodine use for Graves’ patients (23). However, with advancements in pharmacological strategies, the use of energy-based surgical instruments, and improvements in surgical techniques, the safety of thyroid surgery has significantly increased, prompting many physicians to adopt individualized preoperative preparation plans, thus challenging the role of iodine solutions. Hope et al. pointed out that the evidence supporting the preoperative use of Lugol’s solution is insufficient, failing to demonstrate significant improvements in postoperative outcomes (24). A meta-analysis indicated that while preoperative iodine administration can reduce thyroid vascularity and intraoperative blood loss, it does not have a significant impact on operation duration or postoperative complications, and may even prolong surgery time (25). Recent studies have shown that in patients undergoing total or near-total thyroidectomy without iodine preparation, the rates of temporary and permanent recurrent laryngeal nerve injury, temporary and permanent hypoparathyroidism, and thyroid storm incidence are consistent with reported literature rates (25–28). Considering the severity of this patient’s hyperthyroid symptoms, particularly the tracheal compression caused by diffuse goiter, the use of potassium iodide solution (Lugol’s solution) prior to surgery was deemed important. However, a severe complication of thyroid storm occurred postoperatively. This may be attributed to the long-term, high-dose administration of Lugol’s solution increasing the risk of iodine escape, and the gradually hardened texture of the thyroid gland, which, when subjected to surgical pressure, could lead to significant hormone release. Additionally, stress, anesthesia, or surgical maneuvers may also trigger a thyroid storm. Therefore, a thorough preoperative preparation and assessment based on MDT discussions for patients with GD is essential.

Thyroid storm is a severe postoperative complication of hyperthyroidism, typically occurring within 12 to 36 hours after surgery, characterized by rapid progression and a high mortality rate (29). The onset of this crisis is often associated with insufficient preoperative correction of thyroid function, elevated basal metabolic rate, surgical stress response, and adrenal insufficiency. However, whether thyroid function must be normalized preoperatively before surgery remains a matter of debate. A retrospective cohort study evaluating 67 patients with GD who underwent thyroidectomy found that 33% were in a subclinical hyperthyroid state, while 21% exhibited overt hyperthyroidism; yet, none developed thyroid storm postoperatively (30). Similarly, another study analyzing 165 GD patients who underwent total thyroidectomy reported that only 2% received iodine preparation preoperatively, and 42% still had hyperthyroidism during the procedure, but again, no cases of thyroid storm were observed postoperatively (26).Larger-scale cohort studies are needed in the future to further validate these findings.

Parathyroid hyperplasia is one of the primary causes of PHPT (31). Actively removing multiple or all parathyroid glands can effectively reduce the risk of persistent or recurrent hyperparathyroidism, but long-term parathyroid dysfunction can severely impact the patient’s quality of life. Since parathyroid hyperplasia does not occur synchronously, blindly performing a three-and-a-half gland resection is a crude approach. For example, in patients with hyperparathyroidism associated with MEN1, even after the removal of three and a half or more parathyroid glands, 5% to 6% of patients may continue to have the disease (32). Additionally, total parathyroidectomy with autotransplantation carries the risk of irreversible parathyroid hypofunction, particularly in younger patients. Genetic sequencing is crucial for the qualitative diagnosis of PHPT (33), but the patient in our case declined whole genome sequencing. In our case, after resecting the affected left lower parathyroid gland, a significant drop in PTH was observed, while other parathyroid glands were preserved. Although current scientific technology cannot predict the order, speed, and extent of hyperplasia in four or more glands, the widespread use of localization imaging techniques such as choline scanning, the clinical promotion of agents like calcimimetics and RANK inhibitors, as well as the skilled application of minimally invasive techniques like ablation (34), enable the MDT to formulate individualized treatment plans for patients. These plans may include medication management, sequential resection of the dominant gland, additional pharmacotherapy, and if necessary, further contralateral surgery or ablation.

This study has several limitations. Firstly, the follow-up period is relatively short, and long-term recurrence risks, particularly regarding parathyroid function and calcium-phosphorus homeostasis, require continuous monitoring, necessitating further follow-up in future management. Secondly, due to the scarcity of relevant literature, we were able to include only 8 reported cases, which limits the comprehensive understanding of this rare clinical event and the generalizability of the conclusions. Lastly, the patient’s refusal to undergo genetic testing restricted our ability to explore the underlying pathogenic mechanisms. Despite these limitations, our study provides valuable insights into the management of patients with concurrent PH and PHPT. Firstly, we conducted a comprehensive narrative review of the existing literature, systematically synthesizing data from 8 reported cases. To our knowledge, this represents the most extensive compilation of such cases to date. Secondly, our case highlights the importance of multidisciplinary collaboration in preoperative preparation, intraoperative decision-making, and postoperative complication management. Finally, the detailed documentation of the clinical process, from diagnostic challenges to long-term outcomes, provides actionable insights for clinicians. By emphasizing the correlation between hypercalcemia and PHPT in hyperthyroid patients, we reinforce the necessity of routine calcium and PTH screening in this population, which may help reduce diagnostic delays.

## Conclusion

In conclusion, cases of HP combined with PHPT are relatively rare in clinical practice. However, for hyperthyroid patients with hypercalcemia, clinicians should be highly vigilant for the possibility of PHPT. Furthermore, the decision regarding the use of iodine solutions prior to surgery for hyperthyroidism and the extent of surgical removal for parathyroid hyperplasia should be determined through effective preoperative assessment, imaging studies, and collaboration within a MDT. This approach allows for the formulation of individualized treatment plans, minimizing the risks of postoperative thyroid storm, recurrence of hyperparathyroidism, and permanent parathyroid dysfunction.

## Data Availability

The original contributions presented in the study are included in the article/supplementary material. Further inquiries can be directed to the corresponding authors.

## References

[1] Cooper DS. Hyperthyroidism. Lancet. (2003) 362:459–68. doi: 10.1016/s0140-6736(03)14073-1 12927435

[2] Brent GA. Clinical practice. Graves’ disease. N Engl J Med. (2008) 358:2594–605. doi: 10.1056/NEJMcp0801880 18550875

[3] Weetman AP. Graves’ disease. N Engl J Med. (2000) 343:1236–48. doi: 10.1056/nejm200010263431707 11071676

[4] Rizk Y Saad N Arnaout W Chalah MA Farah S. Primary hyperparathyroidism in older adults: A narrative review of the most recent literature on epidemiology, diagnosis and management. J Clin Med. (2023) 12. doi: 10.3390/jcm12196321 PMC10573864 37834965

[5] Golden SH Robinson KA Saldanha I Anton B Ladenson PW. Clinical review: Prevalence and incidence of endocrine and metabolic disorders in the United States: a comprehensive review. J Clin Endocrinol Metab. (2009) 94:1853–78. doi: 10.1210/jc.2008-2291 PMC5393375 19494161

[6] Calò PG Medas F Loi G Pisano G Sorrenti S Erdas E. Parathyroidectomy for primary hyperparathyroidism in the elderly: experience of a single endocrine surgery center. Aging Clin Exp Res. (2017) 29:15–21. doi: 10.1007/s40520-016-0666-7 27837463

[7] Minisola S Gianotti L Bhadada S Silverberg SJ. Classical complications of primary hyperparathyroidism. Best Pract Res Clin Endocrinol Metab. (2018) 32:791–803. doi: 10.1016/j.beem.2018.09.001 30665547

[8] Spanheimer PM Weigel RJ. Management of patients with primary hyperparathyroidism and concurrent thyroid disease: an evolving field. Ann Surg Oncol. (2012) 19:1428–9. doi: 10.1245/s10434-012-2286-6 22395994

[9] Zhang W Liu F Chen K Wang Y Dou J Mu Y. Case report: coexistence of primary hyperparathyroidism with giant toxic nodular goiter. BMC Endocr Disord. (2022) 22:200. doi: 10.1186/s12902-022-01117-0 35945539 PMC9361506

[10] Wagner B Begic-Karup S Raber W Schneider B Waldhäusl W Vierhapper H. Prevalence of primary hyperparathyroidism in 13387 patients with thyroid diseases, newly diagnosed by screening of serum calcium. Exp Clin Endocrinol Diabetes. (1999) 107:457–61. doi: 10.1055/s-0029-1212138 10595598

[11] Castellano E Benso P Attanasio R Boriano A Lauro C Borretta G. Surgical approach to primary hyperparathyroidism in patients with concomitant thyroid diseases: A retrospective single center study. Int J Endocrinol. (2020) 2020:2182539. doi: 10.1155/2020/2182539 32148486 PMC7057020

[12] Abboud B Sleilaty G Mansour E El Ghoul R Tohme C Noun R. Prevalence and risk factors for primary hyperparathyroidism in hyperthyroid patients. Head Neck. (2006) 28:420–6. doi: 10.1002/hed.20366 16388525

[13] Musiałkiewicz J Komarnicki P Ziółkowska P Czepczyński R Ruchała M Gut P. Hyperthyroidism caused by massive toxic nodular goiter accompanied by primary hyperparathyroidism. Pol Arch Intern Med. (2024) 134. doi: 10.20452/pamw.16802 39007670

[14] He Y Liu S Guo H Shi B. Incidental finding of papillary thyroid carcinoma with BRAFV600E mutation in a patient with coexistent primary hyperparathyroidism and Graves’ hyperthyroidism. BMJ Case Rep. (2014) 2014. doi: 10.1136/bcr-2013-203436 PMC4039927 24879726

[15] Yokomoto M Minamoto M Utsunomiya D Umakoshi H Fukuoka T Kondo S. Hypercalcemic crisis due to primary hyperparathyroidism occurring concomitantly with Graves’ disease. Intern Med. (2015) 54:813–8. doi: 10.2169/internalmedicine.54.2605 25832948

[16] Nagaki S Tachikawa E Kodama H Obara T Osawa M Nagata S. A case of Turner’s syndrome with Graves’ disease and primary hyperparathyroidism. SAGE Open Med Case Rep. (2021) 9:2050313x211059002. doi: 10.1177/2050313x211059002 PMC8673862 34925839

[17] Xiao H Yu B Wang S Chen G. Concomitant Graves’ disease and primary hyperparathyroidism: the first case report in mainland of China and literature review. Chin Med J (Engl). (2002) 115:939–41. 12136811

[18] Miani C Bracale AM Bresadola V Motz E. Concomitant primary hyperparathyroidism, Graves’ disease and vitamin D deficiency. Acta Otorhinolaryngol Ital. (2003) 23:199–202. 14677315

[19] Tachibana S Sato S Yokoi T Nagaishi R Akehi Y Yanase T. Severe hypocalcemia complicated by postsurgical hypoparathyroidism and hungry bone syndrome in a patient with primary hyperparathyroidism, Graves’ disease, and acromegaly. Intern Med. (2012) 51:1869–73. doi: 10.2169/internalmedicine.51.7102 22821103

[20] Ho KJ. Papillary parathyroid adenoma. A rare occurrence and its importance in differentiation from papillary carcinoma of the thyroid. Arch Pathol Lab Med. (1996) 120:883–4. 9140297

[21] Padberg B Schröder S Capella C Frilling A Klöppel G Heitz PU. Multiple endocrine neoplasia type 1 (MEN 1) revisited. Virchows Arch. (1995) 426:541–8. doi: 10.1007/bf00192107 7655733

[22] Iqbal AA Burgess EH Gallina DL Nanes MS Cook CB. Hypercalcemia in hyperthyroidism: patterns of serum calcium, parathyroid hormone, and 1,25-dihydroxyvitamin D3 levels during management of thyrotoxicosis. Endocr Pract. (2003) 9:517–21. doi: 10.4158/ep.9.6.517 14715479

[23] Ross DS Burch HB Cooper DS Greenlee MC Laurberg P Maia AL. 2016 American thyroid association guidelines for diagnosis and management of hyperthyroidism and other causes of thyrotoxicosis. Thyroid. (2016) 26:1343–421. doi: 10.1089/thy.2016.0229 27521067

[24] Hope N Kelly A. Pre-operative Lugol’s iodine treatment in the management of patients undergoing thyroidectomy for Graves’ Disease: A review of the literature. Eur Thyroid J. (2017) 6:20–5. doi: 10.1159/000450976 PMC5465802 28611944

[25] Tsai CH Yang PS Lee JJ Liu TP Kuo CY Cheng SP. Effects of preoperative iodine administration on thyroidectomy for hyperthyroidism: A systematic review and meta-analysis. Otolaryngol Head Neck Surg. (2019) 160:993–1002. doi: 10.1177/0194599819829052 30721111

[26] Shinall MC Jr. Broome JT Baker A Solorzano CC. Is potassium iodide solution necessary before total thyroidectomy for Graves disease? Ann Surg Oncol. (2013) 20:2964–7. doi: 10.1245/s10434-013-3126-z 23846785

[27] Hassan I Danila R Aljabri H Hoffmann S Wunderlich A Karakas E. Is rapid preparation for thyroidectomy in severe Graves’ disease beneficial? The relationship between clinical and immunohistochemical aspects. Endocrine. (2008) 33:189–95. doi: 10.1007/s12020-008-9076-8 18493879

[28] Kaur S Parr JH Ramsay ID Hennebry TM Jarvis KJ Lester E. Effect of preoperative iodine in patients with Graves’ disease controlled with antithyroid drugs and thyroxine. Ann R Coll Surg Engl. (1988) 70:123–7. PMC2498739 2457351

[29] Akamizu T. Thyroid storm: A Japanese perspective. Thyroid. (2018) 28:32–40. doi: 10.1089/thy.2017.0243 28899229 PMC5770119

[30] Al Jassim A Wallace T Bouhabel S Majdan A Hier M Forest VI. A retrospective cohort study: do patients with graves’ disease need to be euthyroid prior to surgery? J Otolaryngol Head Neck Surg. (2018) 47:37. doi: 10.1186/s40463-018-0281-z 29784035 PMC5963139

[31] Erickson LA Mete O Juhlin CC Perren A Gill AJ. Overview of the 2022 WHO classification of parathyroid tumors. Endocr Pathol. (2022) 33:64–89. doi: 10.1007/s12022-022-09709-1 35175514

[32] Yavropoulou MP Vlachou S Tsoli M Fostira F Kaltsas G Kassi E. Management and long-term follow-up of hyperparathyroidism in multiple endocrine neoplasia type 1: single center experience. J Clin Med. (2022) 11. doi: 10.3390/jcm11071967 PMC8999236 35407574

[33] Park HS Lee YH Hong N Won D Rhee Y. Germline mutations related to primary hyperparathyroidism identified by next-generation sequencing. Front Endocrinol (Lausanne). (2022) 13:853171. doi: 10.3389/fendo.2022.853171 35586626 PMC9109676

[34] Muhetaer G Liu G Zhang L Jiang H. Severe secondary hyperparathyroidism in a chronic kidney disease patient treated with Radiofrequency ablation: One case report. Front Med (Lausanne). (2022) 9:876692. doi: 10.3389/fmed.2022.876692 35935765 PMC9353393

